# The risk of major cardiovascular events for adults with transfemoral amputation

**DOI:** 10.1186/s12984-018-0400-0

**Published:** 2018-09-05

**Authors:** Benjamin F. Mundell, Marianne T. Luetmer, Hilal Maradit Kremers, Sue Visscher, Kurtis M. Hoppe, Kenton R. Kaufman

**Affiliations:** 1 Mayo Clinic School of Medicine, Mayo Clinic, Rochester, MN USA; 2 Department of Physical Medicine and Rehabilitation, Mayo Clinic, Rochester, MN USA; 3 Department of Orthopedic Surgery, Mayo Clinic, Rochester, MN USA; 4 Department Health Sciences Research, Mayo Clinic, Rochester, MN USA; 5 Center for the Science of Health Care Delivery, Mayo Clinic, Rochester, MN USA

**Keywords:** Transfemoral amputation, Major cardiac event, Competing risk survival analysis

## Abstract

**Background:**

It is well-known that the risk of cardiac disease is increased for those with lower-limb amputations, likely as a result of the etiology of the amputation. Using a longitudinal population-based dataset, we examined the association between transfemoral amputation (TFA) status and the risk of experiencing a major cardiac event for those undergoing either dysvascular or traumatic amputations. The association of receiving a prosthesis with the risk of experiencing a major cardiac event was also examined.

**Methods:**

*Study Population*: All individuals with TFA (N 162), i.e. knee disarticulation and transfemoral amputation, residing in Olmsted County, MN, between 1987 and 2014. Each was matched (1:10 ratio) with non-TFA adults on age, sex, and duration of residency.

*Data Analysis*: A competing risk Cox proportional hazard model was used to estimate the relative likelihood of an individual with a TFA experiencing a major cardiac event in a given time period as compared to the matched controls. The cohort was divided by amputation etiology: dysvascular vs trauma/cancer. Additional analysis was performed by combining all individuals with a TFA to look at the relationship between prosthesis receipt and major cardiac events.

**Results:**

Individuals with a dysvascular TFA had an approximately four-fold increased risk of a cardiac event after undergoing an amputation (HR 3.78, 95%CI: 3.07–4.49). These individuals also had an increased risk for non-cardiac mortality (HR 6.27, 95%CI: 6.11–6.58). The risk of a cardiac event was no higher for those with a trauma/cancer TFA relative to the able-bodied controls (HR 1.30, 95%CI: 0.30–5.85). Finally, there was no difference in risk of experiencing a cardiac event for those with or without prosthesis (HR 1.20, 95%CI: 0.55–2.62).

**Conclusion:**

The high risk of initial mortality stemming from an amputation event may preclude many amputees from cardiovascular disease progression. Amputation etiology is also an important factor: cardiac events appear to be more likely among patients with a dysvascular TFA. Providing a prosthesis does not appear to be associated with a reduced risk of a major cardiac event following amputation.

## Background

Individuals with amputations due to dysvascular causes are at increased risk of cardiovascular disease, which is associated with increased peri- and post-operative mortality [1–3], is one of the leading causes of death [4], and is associated with increased disability. [2, 5] In 2015, there were two million Americans living with limb loss, most commonly due to diabetes and peripheral arterial disease [1]. The age-adjusted rate of transfemoral amputation (TFA) reaches 40 per 100,000 patients with diabetes [6]. Due in part to the aging population and increase in prevalence of those living with diabetes, the number of American amputees is projected to double by the year 2050. [7, 8]

This growing population of dysvascular amputees has a higher prevalence of cardiovascular disease than the general U.S. adult population: up to 75% have coronary artery disease, 60–80% have hypertension, 15–25% have cerebrovascular disease, and 20–50% have congestive heart failure. [4, 9–12] In comparison, only approximately 37% of U.S. adults have at least one type of cardiovascular disease. [13] Cardiovascular disease remains the leading cause of death and health care expenditure in the U.S., with direct and indirect costs reaching $316.1 billion between 2012 and 2013 [13]. Expenditures are expected to nearly triple by the year 2030, with a large proportion of these growing costs attributable to modifiable risk factors [13–16].

To date studies evaluating major cardiovascular events (MACE), including cardiac death or non-fatal myocardial infarction among individuals with TFA, have been cross-sectional. There has not been a longitudinal evaluation of MACE risk in a population-based dataset. This study was undertaken to examine the association between TFA status and the long-term risk of experiencing a major cardiac event for those who underwent an amputation due to either a dysvascular or traumatic cause. The association between receiving a prosthesis and the risk of experiencing a major cardiac event was also examined.

## Methods

### Data source and study population

Individuals with TFA residing in Olmsted County, MN, were identified using the resources available through the Rochester Epidemiology Project (REP). The REP was designed to take advantage of the unique circumstances within Olmsted County: being relatively isolated from other urban areas and having only a few health care providers including Mayo Clinic, Olmsted Medical Center, and their affiliates. [17] The Olmsted County population is similar to that of the Upper Midwest but is less diverse, wealthier, and more highly educated than the general U.S. population, yet results have been found to be generalizable to populations outside the Upper Midwest. [18]

Using the resources of the REP, TFA (both incident and prevalent TFA patients) were identified using the ICD-9 diagnostic & procedure codes for amputations (84.17 for a TFA procedure or V49.76 indication and individual has a TFA). Each adult with TFA was matched (1:10 ratio) with adults without TFA on age, sex, and duration of residency in Olmsted County. All of the controls also resided in Olmsted County during the same period as the individuals with TFA and were identified in the REP. The 10:1 matching was used to capture the largest possible representative sample of controls and it allowed for an equal number of controls per subject. Patients who had denied research authorization for use of their medical records in research were excluded. This study was approved by both the Mayo Clinic and Olmsted Medical Center Institutional Review Boards.

Medical records for TFA individuals were reviewed to confirm their amputation status and level. Additional data obtained included gender, race, amputation etiology, year of amputation (index date), pre and post-amputation comorbidities, and use of prosthesis. Comorbidities were extracted from administrative data and classified using modified Charlson comorbidities via the *icd9* package in R [19, 20]. The outcome of interest was whether an individual had a major cardiovascular event (MACE), defined as cardiac death, non-fatal myocardial infarction, or coronary revascularization, while residing in Olmsted County. Events were identified using pre-existing REP scripts for MACE including Berkson, Hospital Adaptation of the International Classification of Diseases (HICDA), and ICD-9/− 10 diagnostic codes.

### Statistical analysis

Due to the relatively high initial rates of mortality among individuals with TFA, a competing risk Cox proportional hazard model was used that account for the risk of death due to other cause and the risk of experiencing a MACE in a given time period [21]. The relative risk (hazard ratio) of individuals with a TFA experiencing a MACE was compared to matched controls. The cohort was divided by amputation etiology: dysvascular vs trauma/cancer. Additional analysis was performed on only TFA to look at the relationship between prosthesis receipt and MACE. Because the prosthesis receipt occurs after the index date for most subjects, prosthesis status was treated at a time-dependent covariate. Differences throughout all of the analysis were considered statistically significant at *p* < .05. Simulated survival curves for each sub-cohort were performed for a man of average age and Charlson Comorbidity Index value to provide a visualization of model results. All statistical analysis was conducted using R version 3.3.2. [22]

## Results

The study population included at total of 162 individuals with TFA; 107 with amputation due to dysvascular etiology and 55 secondary to trauma or cancer. Mean age at amputation was 75.7 ± 11.3 years for those with dysvascular disease and 32.5 ± 20.8 years for those with TFA due to trauma or cancer (*p* < 0.001). Men with dysvascular TFA were significantly younger at time of amputation than females with dysvascular TFA (*p* = 0.020, Table 1). Those with dysvascular amputations had a significantly higher mortality rate at 1 and 5 years compared to those with TFA secondary to trauma or cancer (*p* < 0.001, Table 1).

**Table 1:** Demographic information

Amputation Etiology	Total	Men (number (%))	Mean Age at Amputation Years (SD)		Mortality Rate (%)		*p* value
			Men	Women	1 year	5 year	
Dysvascular	107	55 (51.4%)	73.27 (10.7)	78.33 (11.4)	28%	45%	0.020
Trauma/Cancer	55	39 (70.9%)	31.57 (19.3)	34.57 (24.6)	2%	2%	0.631
All	162	94 (58.0%)	55.97 (25.4)	68.04 (24.2)	19%	30%	0.003

Patients with a dysvascular TFA were more likely to have MACE compared to patients with amputations due to trauma or cancer, with the exception of coronary stent placement (Table 2). Both the patients with a TFA due to dysvascular etiology and those due to either trauma or cancer had greater rates of myocardial infarctions when compared to their respective matched non-TFA cohorts. Overall, however, patients with TFA were no more likely to experience a MACE than their respective controls.

**Table 2:** Incidence of major cardiovascular events (MACE) following a transfemoral amputation

Amputation Etiology	Cohort	MACE total (%)	Coronary artery bypass Graft (%)	Ischemic heart disease (%)	Myocardial infarction (%)	Percutaneous transluminal coronary angioplasty (%)	Coronary stent placement (%)	Cardiac arrest (%)
Dysvascular	TFA, n = 107	71 (66.4)	3 (4.2)	7 (9.9)	52 (73.2)	2 (2.8)	0 (0)	7 (9.9)
	Controls, *n* = 1070	423 (39.5)	23 (5.4)	110 (26.0)	235 (55.6)	12 (2.8)	2 (0.5)	41 (9.7)
Trauma/Cancer	TFA, n = 55	11 (20.0)	1 (9.1)	1 (9.1)	7 (63.6)	1 (9.1)	0 (0)	1 (9.1)
	Controls, *n* = 550	88 (16.0)	4 (4.5)	17 (19.3)	46 (5.2)	8 (9.1)	0 (0)	13 (14.8)

NB: Events and percentages represent the respective proportion of those individuals experiencing a MACE

### Dysvascular TFA, *N* = 107

**Fig. 1:**
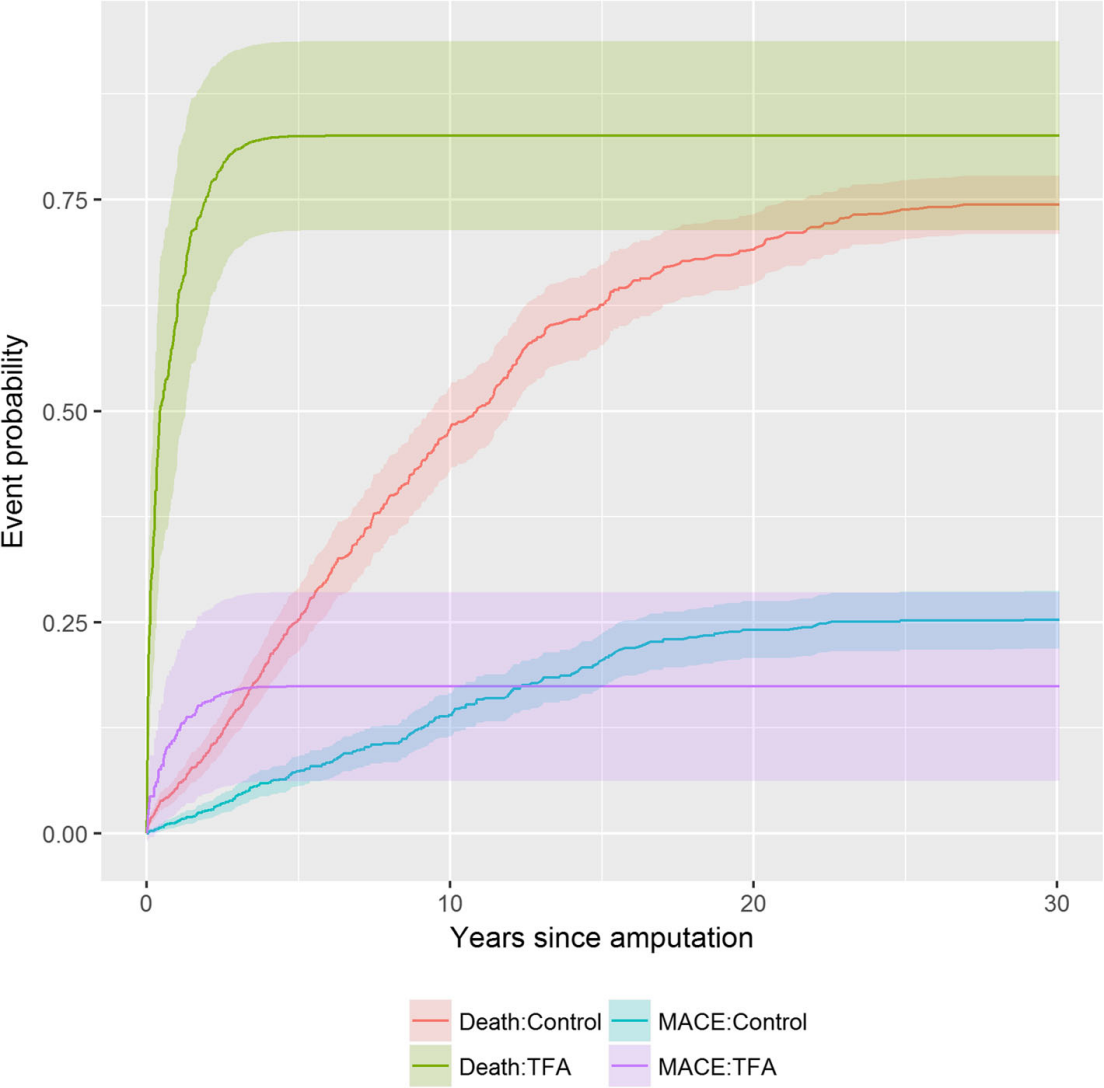
Time dependent probability of a major cardiovascular event (MACE) or death for individuals with a transfemoral amputation due to *dysvascular disease* compared to matched control subjects without an amputation

The early and later risk of mortality appeared to change around 2.5 years following an dysvascular TFA as observed in the initial Kaplan-Meir curves (Fig. 1), therefore a time dependent variable was added to account for this change and satisfy the proportional hazard assumption. Having a dysvascular TFA was associated with an approximately four-fold increase in experiencing a cardiac event both prior to and after 2.5 years of undergoing an amputation (Hazard Ratio (HR) 3.78, 95% CI: 3.07–4.49; HR 4.17, 95% CI: 3.46–4.86). There was also an increased risk for non-cardiac mortality both prior to and after 2.5 years (HR 6.27, 95% CI: 6.11–6.58; HR 3.03, 95% CI: 2.60–3.46) (Fig. 1).

### TFA due to trauma or cancer, *N* = 55

**Fig. 2:**
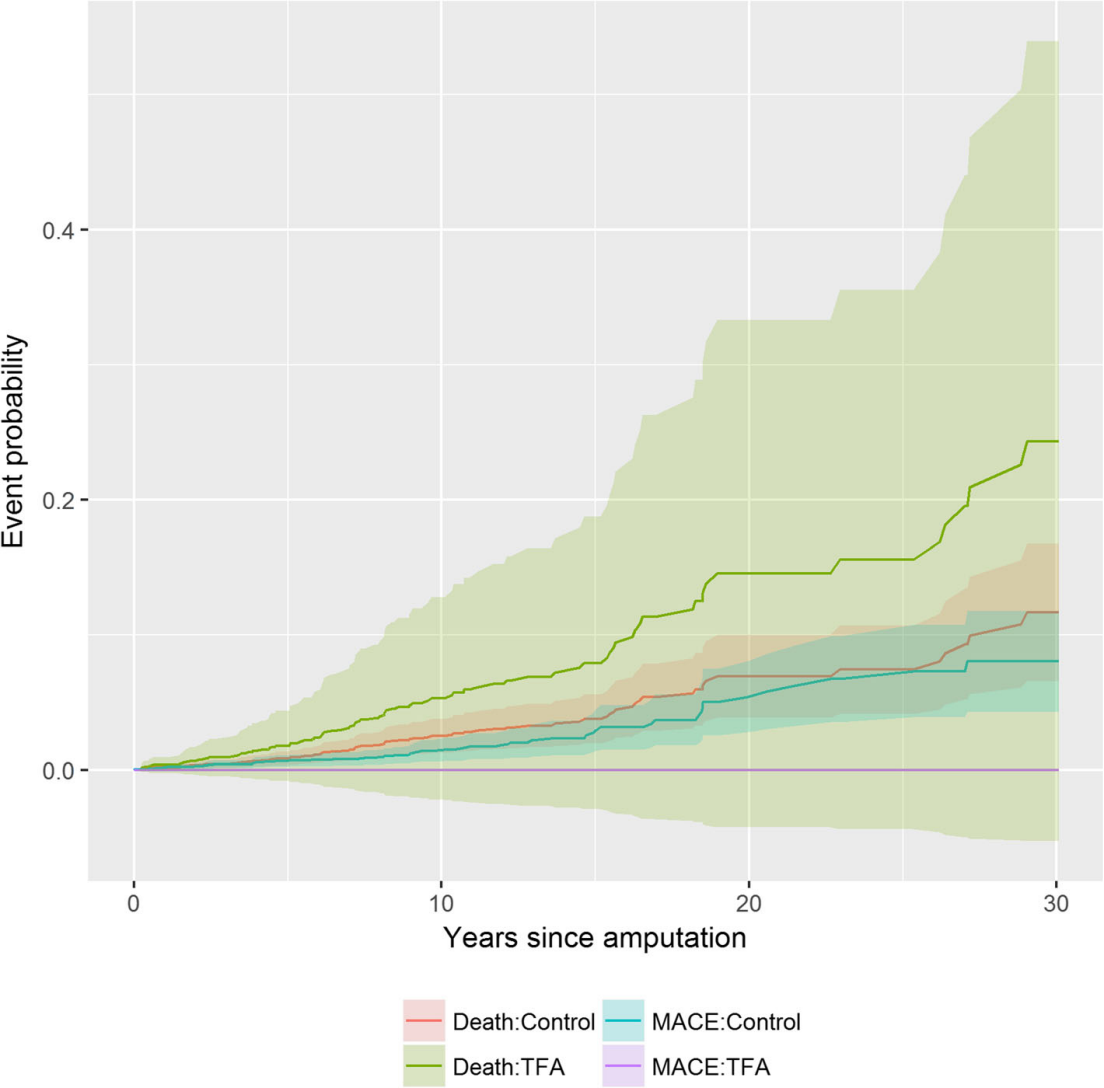
Time dependent probability of a major cardiovascular event (MACE) or death for individuals with a transfemoral amputation due to *trauma or cancer* compared to matched control subjects without an amputation

The early and later risk of mortality appeared to change around 10 years following an trauma or cancer related TFA as observed in the initial Kaplan-Meir curves (Fig. 2), therefore a time dependent variable was added to account for this change and satisfy the proportional hazard assumption. Those with a TFA had no significant increase in experiencing a cardiac event within 10 years or beyond 10 years relative to the controls (HR 1.30, 95% CI: 0.30–5.85; HR 1.60, 95% CI: 0.67–3.80). Adjusted non-cardiac mortality risks did not appear to differ from the controls (HR 1.94, 95% CI: 0.54–6.91; HR 1.45, 95%CI: 0.72–2.93). Individuals with a TFA do not differ in risk of MACE or death compared to control subjects.

### TFA (Dysvascular and trauma/Cancer) with prosthesis, *N* = 70

**Fig. 3:**
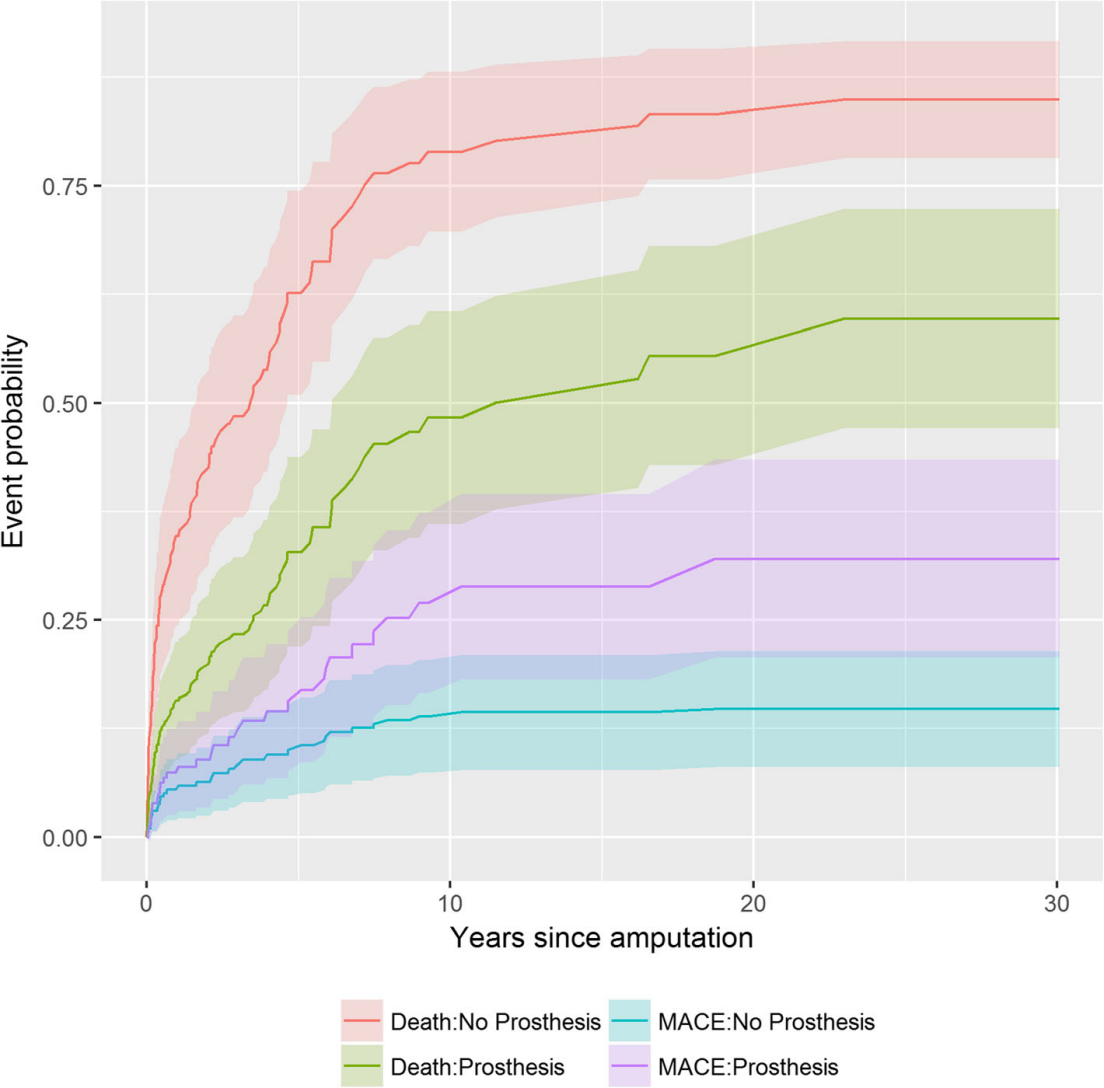
Time dependent probability of a major cardiovascular event (MACE) or death for individuals with a transfemoral amputation who received a prosthesis compared to those who did not receive a prosthesis

Those receiving a prosthesis had almost a 60% reduction in risk of death (HR 0.40, 95% CI: 0.26–0.64). There was no difference in risk of experiencing a major cardiac event for those with or without a prosthesis (HR 1.20, 95% CI: 0.55–2.62) (Fig. 3). The only covariates associated with an increased risk of MACE were age at time of amputation (HR 1.02, 95% CI: 1.01–1.03) and a higher Charlson Comorbidity index (HR 1.25, 95% CI: 1.17–1.33).

## Discussion

This study determined the longitudinal risk of major cardiovascular events (MACE) in patients with an transfemoral amputation. Unlike previous studies, which only evaluated cardiovascular events throughout the perioperative time period or up to 5 years post amputation [9, 10, 23], this study uniquely calculated the risk as a function of time over a 30 year time span in a well characterized population-based observational study. This unique, population-based study suggests that the high risk of initial mortality stemming from an amputation event may preclude many amputees from progression of cardiovascular disease. Mortality rate among individuals with dysvascular TFA in our study was 28% at 1 year and 45% at 5 years, which is lower than previously reported ranges of 43–54% mortality at 1 year and 40–90% at 5 years [11, 24, 25]. Those with dysvascular amputations had a higher all-cause mortality rate than those with TFA secondary to trauma or cancer, which is also consistent with the literature. [5, 23, 25–27] Etiology of the amputation is also an important factor, as MACE were more likely among patients with a dysvascular TFA.

Dysvascular disease progression and need for amputation is a significant risk factor for MACE, and studies show that level of amputation is also important. Mohammedi et al. followed 11,140 patients with diabetes over a median time period of 9.9 years and found that compared to those without peripheral artery disease, those with lower extremity ulceration and or amputation were at a significantly higher risk of cardiovascular death, myocardial infarction, and all-cause mortality (HR 1.91, 1.50, and 1.39 respectively). [28] They did not perform separate risk analyses for patients with ulceration and amputation which is likely why reported risk factors were lower compared to those in our study. Perioperative cardiac event rate is reported to be higher among individuals undergoing TFA compared to those undergoing transtibial amputation, 6.8 and 3.6% respectively. [23]

All-cause mortality as well as the risk of mortality due to cardiovascular disease in patients with transfemoral amputation has been well studied in the military population. Hrubec and Ryder showed that after 15 years, the mortality rate in soldiers with a TFA was significantly higher than in the general population and in veterans with limb preservation [29]. They also showed that soldiers with bilateral TFA have a 3.5X relative risk of mortality compared to veterans who had limb salvage procedures [29]. Modan et al. evaluated the 24-year mortality rates of male traumatic lower limb amputees (*n* = 201) of the Israeli army compared with a cohort sample representing the general population (*n* = 1832) and found that mortality rates were significantly higher (21.9% vs 12.1%) in military amputees than in able-bodied controls [30]. Cardiovascular disease mortality was twice as likely in amputees compared to controls and was cited as the main cause for this difference. Our study differs from the findings reported by Modan (1998), since the adjusted cardiac mortality risk in the patients with TFA due to trauma or cancer did not differ from controls. Although they did not further analyze specific causes of mortality within cardiovascular disease, they reported a trend towards an increase in the risk of myocardial infarction among the amputees. This is similar to our finding that those with TFA due to trauma or cancer were more likely to have a myocardial infarction when compared to able-bodied controls.

Several studies have revealed that only about a quarter of individuals with transfemoral amputations receive a prosthesis [31–34] A 10-year increase in age in the civilian population has been shown to result in a 54% decrease in the likelihood of being fit for a prosthesis [35]. Similarly, a study of elderly US veterans revealed that a 10-year age increase reduced the likelihood of receiving a prosthesis by 78% [36]. Relatedly, the odds of receiving a prosthesis were almost 30 times higher in those able to walk independently prior to an amputation relative to those who could not walk independently [35]. Interestingly, time elapsed between surgery and the prosthesis decision was associated with a rise in the probability of receiving a prosthesis for the first 3 months after the amputation [35]. These data as a whole illustrate the lack of consistent, reliable prosthesis prescriptions and treatment, and the unnecessary variability of care that patients with limb loss currently receive. While the data in this study appeared to show that receipt of a prosthesis was correlated with a decreased mortality risk from non-cardiac events, this was likely due to the fact that to receive a prosthesis, one had to live for some time following a TFA and therefore it is unlikely due to a protective effect from the prosthesis. Due to limitations in the data, it was difficult to account for this endogeneity. Notably, receipt of a prosthesis does not appear to be associated with a reduced risk of a major cardiac event following amputation.

## Conclusion

This unique, population-based study suggests that the high risk of initial mortality stemming from an amputation event may preclude many amputees with dysvascular disease from progression of cardiovascular disease. In contrast, patient who had an amputation due to trauma or cancer have no greater risk of a cardiovascular event than individuals without an amputation. Notably, prosthesis receipt was not associated with a decreased risk of experiencing a MACE.
